# Bilateral vertebral artery injury leads to brain death following traumatic brain injury: a case report

**DOI:** 10.1186/s13256-024-04432-3

**Published:** 2024-03-16

**Authors:** Vera Irawany, Vizzi A. F. Nasution, Noorcahya Amalia

**Affiliations:** 1 Fatmawati National General Hospital, Jakarta, Indonesia; 2 Anesthesiology and Intensive Care Department, Faculty of Medicine, Universitas Indonesia, Jakarta, Indonesia

**Keywords:** Cerebrovascular trauma, Carotid artery injury, Digital subtraction angiography, Spondylolisthesis, Cervical vertebrae

## Abstract

**Background:**

Vertebral artery injury is a rare condition in trauma settings. In the advanced stages, it causes death.

**Case:**

A 31-year-old Sundanese woman with cerebral edema, C2–C3 anterolisthesis, and Le Fort III fracture after a motorcycle accident was admitted to the emergency room. On the fifth day, she underwent arch bar maxillomandibular application and debridement in general anesthesia with a hyperextended neck position. Unfortunately, her rigid neck collar was removed in the high care unit before surgery. Her condition deteriorated 72 hours after surgery. Digital subtraction angiography revealed a grade 5 bilateral vertebral artery injury due to cervical spine displacement and a grade 4 left internal carotid artery injury with a carotid cavernous fistula (CCF). The patient was declared brain death as not improved cerebral perfusion after CCF coiling.

**Conclusions:**

Brain death due to cerebral hypoperfusion following cerebrovascular injury in this patient could be prevented by early endovascular intervention and cervical immobilisation.

## Background

Vertebral artery injury following trauma is a rare case with incidence from 0.5 to 2% of all trauma cases. Traumatic vertebral artery injury (TVAI) can be related to cervical spine injury with some mechanisms, such as hyperflexion, hyperextension, distraction, facet dislocation and fractures of the cervical spine. The most related etiology for those injuries are motor vehicle accidents, while the other causes are direct assault, hanging, sports injuries (for example, swimming), and neck manipulation by chiropractors and physiotherapists [1–3].

Symptoms related to TVAI occur in 70% of cases within the first 24 hours post accidents. Other patients may have asymptomatic features or delayed presentation that may lead to undetected late deterioration. Physical findings of posterior circulation ischaemia include dysarthria, impaired balance and coordination, ataxic gait, visual field defects, diplopia, nystagmus, Horner’s syndrome, hiccups, lateral or medial medullary syndrome, lower cranial nerve palsies, papillary abnormalities and impaired consciousness. Due to the high proportion of asymptomatic cases, Denver criteria that consist of signs, symptoms, and risk factors of TVAI can be used as screening tools. Digital subtraction angiography (DSA), CT, or MR angiography are radiology modalities for confirming the diagnosis [1, 4, 5].

Treatment options for TVAI consist of observation, anticoagulation, endovascular treatment, and surgery. Heparin followed by warfarin for three months can be given as a conventional strategy. Open surgical treatment may be considered in uncontrolled hemorrhage [1].

The mortality rate of TVAI varies in the range of 11–100% based on disease stages. This paper describes a rare case of bilateral vertebral injury leading to brain death after traumatic brain injury.

## Case report

**Fig. 1:**
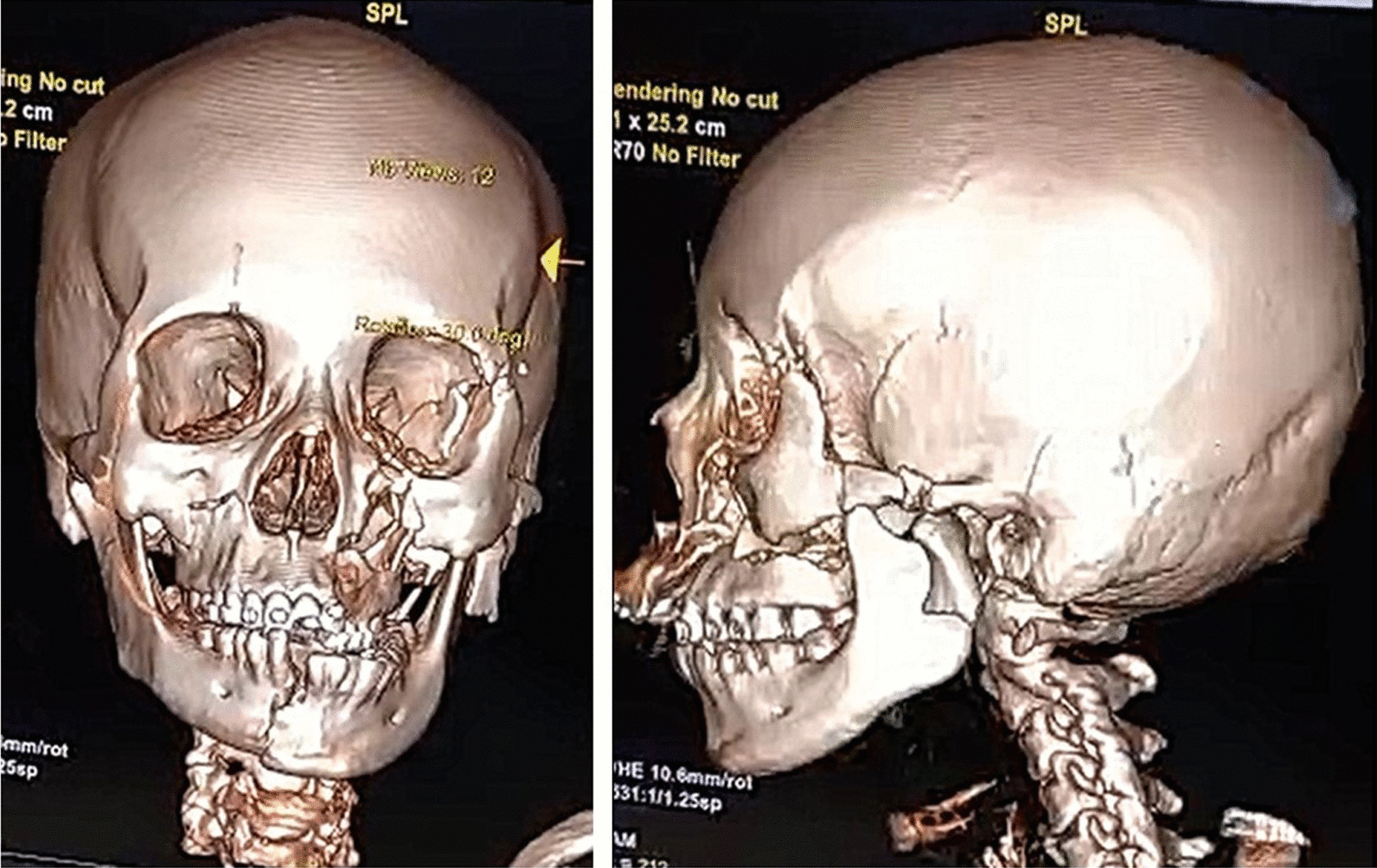
Head computerized tomography showed Le Fort III fracture

**Fig. 2:**
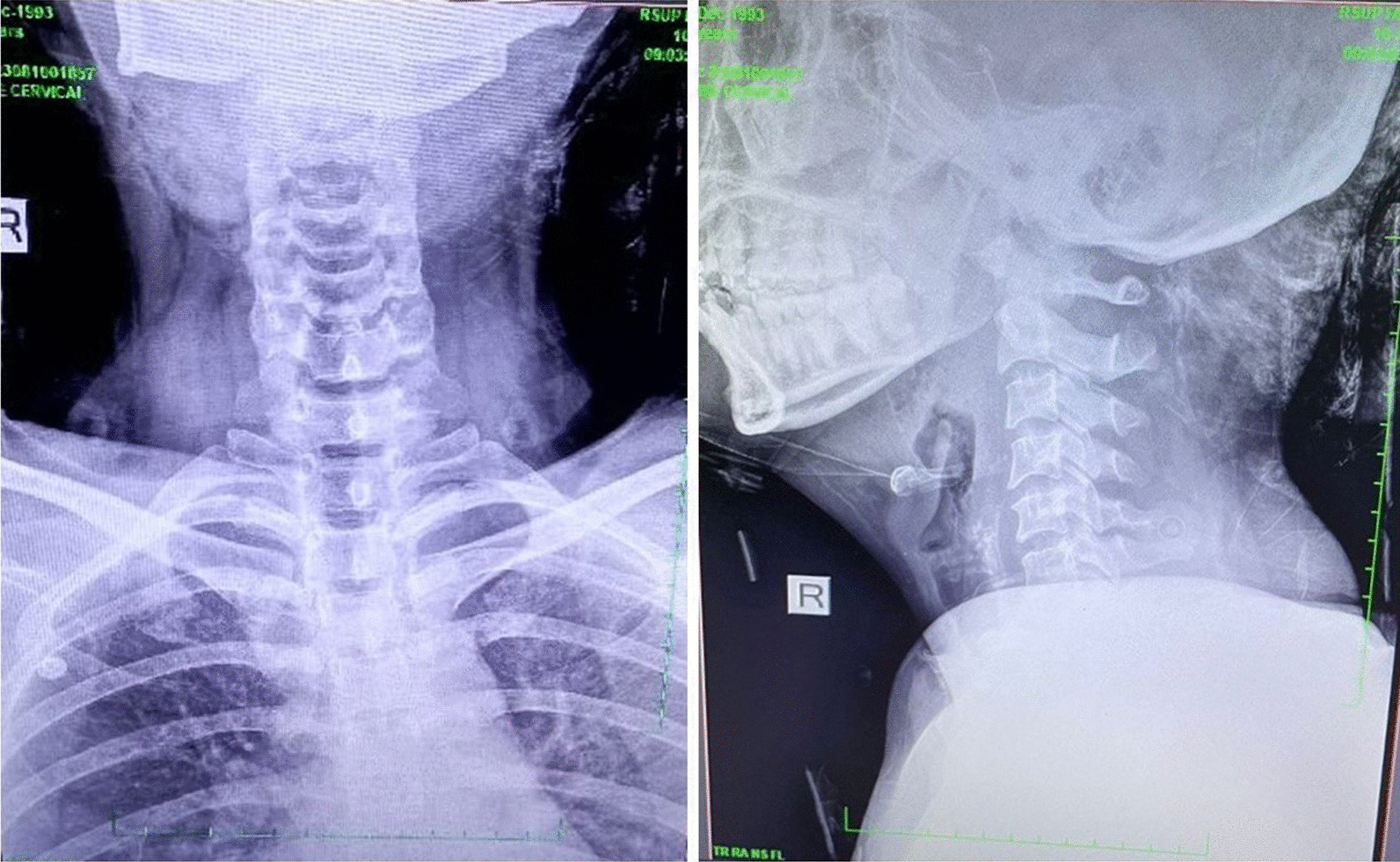
Cervical X-ray showed grade 1 C2–C3 anterolysthesis with prevertebra soft tissue swelling, suspected hematoma

A Sundanese female, 31 years old, was admitted to the emergency room following a motorcycle accident. Her Glasgow Coma Scale (GCS) was 12 with isochoric pupils, normal pupillary light reflex, and without another neurologic deficit. Other vital signs were within normal limits. Immediate head computed tomography (CT) showed Le Fort III fracture with cerebral edema (Fig. 1). There were grade 1 C2–C3 anterolisthesis with pre-vertebra soft tissue swelling suspected hematoma in the cervical X-ray result (Fig. 2).

The patient was admitted to the high care unit (HCU) with a rigid collar neck and scheduled elective arch bar maxillomandibular application and debridement. The patient was assessed by Neurology with right extremities weakness and positive right pathologic reflex. The patient was planned to have cervical magnetic resonance imaging but was cancelled due to her agitated situation. The rigid neck collar was removed after five days in HCU.

**Fig. 3:**
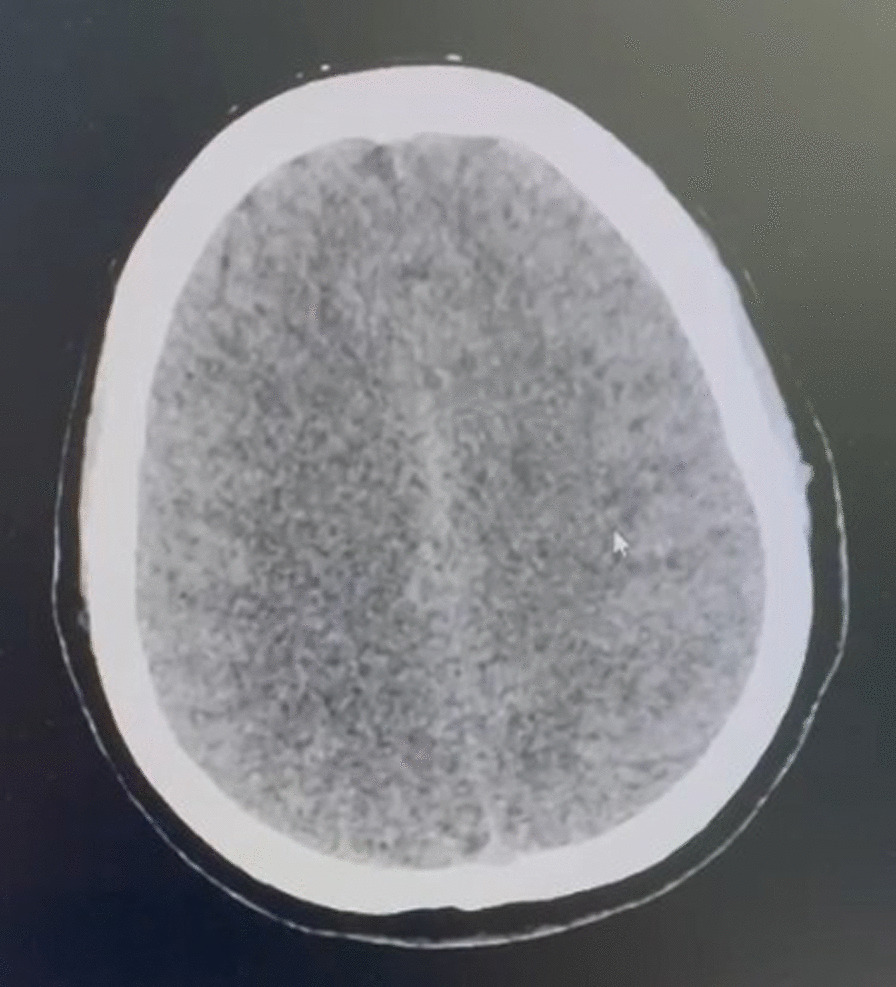
Head computerized tomography evaluation showed bilateral centrum ovale infarction (shown by arrow)

She underwent arch bar maxillomandibular application and debridement on the fifth day in general anesthesia with a hyperextended neck position and nasotracheal tube during the procedure. She was admitted to the intensive care unit (ICU) post procedure. We used thiopental as a sedative agent to decrease intracranial pressure. After 19 hours of monitoring, her right extremities weakness was increased. CT evaluation revealed bilateral centrum ovale infarction (Fig. 3).

**Fig. 4:**
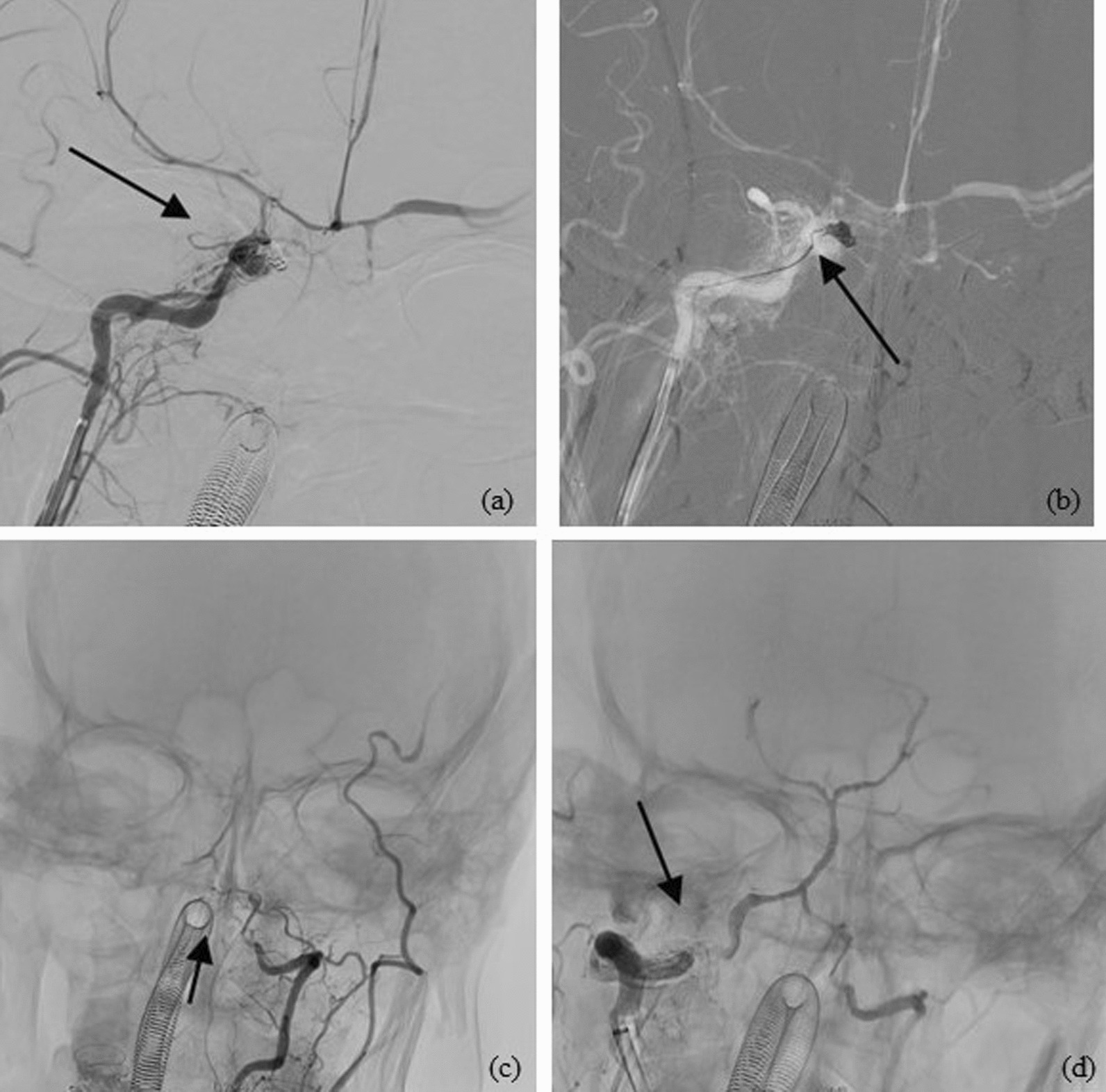
Digital subtraction angiography showed grade 5 left internal carotid artery injury (**a**) with post coiling carotid cavernous fistula (**b**) and grade 5 left (**c**) and right (**d**) vertebral arteries injury (shown by arrow)

**Fig. 5:**
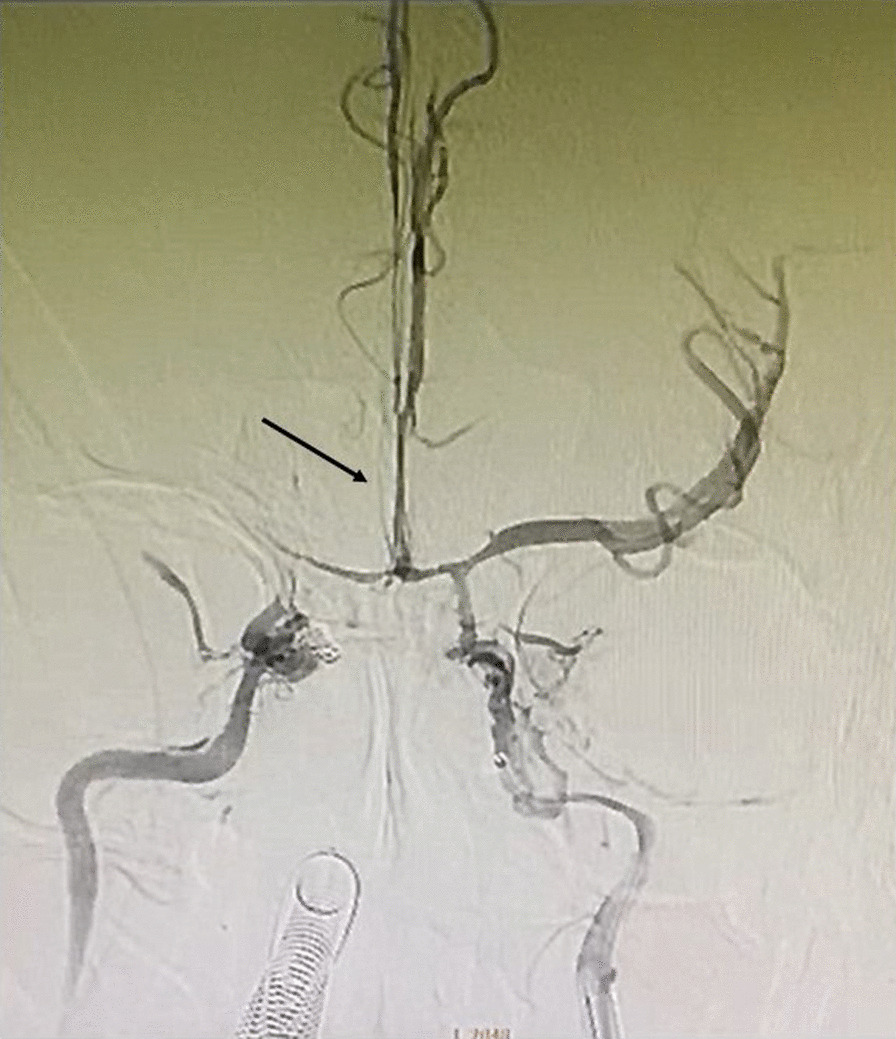
Severe vasospasm appearance (shown by arrow) after carotid cavernosus fistula coiling

Her condition deteriorated on the third day in the ICU. GCS was three without light pupillary reflex. Sedation and analgetic discontinuation did not improve her consciousness. The patient then underwent digital subtraction angiography (DSA). Grade 5 (transection) bilateral vertebral artery injury due to cervical spine displacement and grade 4 (occlusion) left internal carotid artery injury with CCF were recognised during angiography (Fig. 4). CCF coiling was performed, but her cerebral perfusion was not improved with severe vasospasm appearance (Fig. 5). Patient was declared as brain death.

## Discussion

Traumatic vertebral artery injury is a rare case with incidence from 0.5 to 2% of all trauma cases. In this case, cerebrovascular injury was associated with head and neck trauma. Cervical hyperflexion, hyperextension, dislocation, and fracture can cause intramural thrombus formation due to intimal injury leading to total occlusion. In the advanced stage, blood vessel transection, as happened in our patient, can be fatal death (Table 1) [1, 6].

**Table 1:** Cerebrovascular injury classification

Stage		Stroke rate (%)	Mortality rate (%)
1	Lumen narrowing < 25%	3	11
2	Lumen narrowing ≥ 25%, intraluminal thrombus	11	11
3	Pseudoaneurysm	33	11
4	Occlusion	44	22
5	Vessel transection	100	100

According to Denver screening criteria, our patient’s condition is consistent with cerebrovascular injury signs and symptoms (Table 2) [7]. We believe the left internal carotid artery occlusion happened after a head impact. Decreased blood flow due to CCF formation also promoted thrombosis intravascular. Furthermore, vertebral artery injury might have already occurred due to anterolisthesis induced by traumatic brain injury in this patient and worsened after the removal of cervical immobilisation and hyperextension neck position during surgery.

**Table 2:** Denver criteria for cerebrovascular injury screening

Signs and symptoms	Risk factors
Arterial hemorrhage	High-energy transfer mechanism with Le Forte II or III fracture
Cervical bruit	Cervical spine fracture patterns: subluxation, fractures extending into the transverse foramen, fractures of C1–C3 vertebrae
Expanding cervical hematoma	Basilar skull fracture with carotid canal involvement
Focal neurologic deficit	Diffuse axonal injury with GCS score
Findings from neurological examination incongruous with head CT scan findings	Near hanging with anoxic brain injury
Stroke on secondary CT scan	

*CT* computerized tomography, *GCS* Glasgow Coma Scale

DSA is a gold standard for diagnosing cerebrovascular injury. Other diagnostic modalities are ultrasonography doppler, magnetic resonance angiography, and computed tomography angiography (CTA) [1, 8]. The treatment strategy includes conservative, endovascular, and surgery based on injury stages [9]. Grade 5 cerebrovascular injury in our patient was indicated to have surgery. Unfortunately, she was already in brain death.

DSA procedure is also the gold standard for diagnosing CCF. CCF closure target is increasing blood flow in the internal carotid artery [10]. However, inadequate intracerebral blood flow in this patient after CCF coiling was aggravated by bilateral vertebral artery injury.

## Conclusion

Brain death in this patient happened due to cerebral hypoperfusion following grade 5 bilateral vertebral artery injury and grade 4 left internal carotid artery injury. We believe those injuries could be prevented using cervical immobilisation and early endovascular intervention. Semirigid immobilisation with a cervical orthosis for 6–12 weeks is a conservative strategy for traumatic spondylolisthesis [11].

## Data Availability

The data sets used during the current study are available from the corresponding author on reasonable request.

## Abbreviations

CCF: Carotid cavernous fistula
GCS: Glasgow Coma Scale
CT: Computed tomography
HCU: High care unit
ICU: Intensive care unit
DSA: Digital subtraction angiography

## Declarations

No institutional review board is needed as there is no direct patient intervention.
